# Suppression of cathepsin B attenuates myocardial injury via limiting cardiomyocyte apoptosis

**DOI:** 10.1515/med-2024-1115

**Published:** 2025-11-26

**Authors:** Ruilin Su, Zhengyang Sun, Tao Chen, Haiqiang Wu, Zhongan Li, Ziyu Tang, Jianbo Zhao, Li Xu

**Affiliations:** Clinical Research Institute of Zhanjiang, Central People’s Hospital of Zhanjiang, Guangdong Medical University, Zhanjiang, Guangdong, 524045, China; Zhanjiang Engineering Technology Research Center of Cerebrovascular Disease Precision Diagnosis and Treatment, Central People’s Hospital of Zhanjiang, Guangdong Medical University, Zhanjiang, Guangdong, 524045, China; Department of Cardiovascular Surgery, Baoan People’s Hospital, Shenzhen, Guangdong, 518100, China; Division of Vascular and Interventional Radiology, Department of General Surgery, Nanfang Hospital, Southern Medical University, Guangzhou, Guangdong, 510515, China

**Keywords:** cathepsin B, Ca074-Me, myocardial injury, cardiomyocyte apoptosis, hypoxia

## Abstract

**Background:**

Inflammation plays a pivotal role in modulating the pathophysiological progression of myocardial injury and the subsequent repair and remodeling of the infarcted myocardium. Cathepsin B, a member of the cysteine protease family, has been recognized for its ability to initiate various signaling cascades essential to inflammatory processes. This study aims to investigate whether cathepsin B influences cardiomyocyte survival under inflammatory conditions.

**Methods:**

Mice were randomly divided into four groups (*n* = 6 per group) based on whether they received an intraperitoneal injection of Ca-074 Me (50 mg/kg) and whether myocardial ischemia/reperfusion (I/R) surgery was performed.

**Results:**

The cathepsin B-specific inhibitor Ca-074 Me significantly attenuated myocardial infarction caused by I/R *in vivo* and reduced hypoxia-induced cardiomyocyte apoptosis *in vitro*. Mechanistically, Ca-074 Me appeared to inhibit caspase-3 signaling, thereby mitigating cardiomyocyte apoptosis under chemical hypoxia induced by cobalt chloride or physical oxygen deprivation.

**Conclusion:**

Targeted inhibition of cathepsin B may represent an innovative strategy for the amelioration of myocardial injury.

## 1 Introduction

Ischemic heart disease (IHD) is a prevalent cardiovascular condition associated with significant morbidity and mortality worldwide [1]. It is characterized by diminished myocardial perfusion that precipitates ischemic damage and subsequent injury to the cardiac muscle [1,2]. The underlying pathophysiology of IHD is complex and multifactorial, with a range of risk factors, including long-term high blood pressure, hyperlipidemia, smoking, and physical activity. The mechanistic basis of myocardial injury stemming from ischemia–reperfusion (I/R) events is relatively complex, encompassing aberrant production of reactive oxygen species, calcium dyshomeostasis, amplification of proinflammatory cytokines, and depletion of high-energy phosphate compounds [3]. Cardiomyocyte apoptosis has been acknowledged as a principal contributor to I/R injury. Accordingly, the exploration of anti-apoptotic modalities harbors therapeutic promise for IHD. Accumulating evidence indicates the inflammatory response triggered by I/R injury plays a significant role in the development and progression of IHD [3,4]. Numerous studies have demonstrated elevated levels of inflammatory mediators (i.e., C-reactive protein, IL-6, and TNF-α) in patients with IHD [4–6]. Clinical trials have exhibited that anti-inflammatory agents, such as monoclonal antibodies against IL-1β, can reduce the risk of cardiovascular events in IHD patients [7]. However, the relationship between inflammation and IHD is complex, and studies elucidating the role and function of inflammatory mediators in cardiomyocyte apoptosis might provide new insights into further directions for IHD.

Cathepsins are proteases with serine, cysteine, or aspartic acid residues as the nucleophiles, which are essential for a range of functions, including digestion, coagulation, immune response, adipogenesis, hormone release, and peptide synthesis [8]. Several studies have shown that cathepsins are involved in the regulation of inflammation [9,10]. For example, cathepsin B has been shown to activate the NLRP3 inflammasome, a key mediator of inflammation, by cleaving pro-IL-1β and pro-IL-18 [11]. During acute pancreatitis, excessive release of cathepsin B into the cytoplasm can lead to cell necrosis through a mechanism dependent on active trypsin [12]. Deletion of cathepsin B alleviated hepatic pathology and enhanced survival in mice with acute liver injury [13]. Cathepsin B seems to degrade a cysteine protease that could eliminate apoptosis, supporting Bax in the cells [14]. Notably, cathepsin B participates in hypoxia-induced disease, and administering cathepsin B inhibitors before ischemia induction can reduce post-ischemic apoptosis of hepatocytes, thereby minimizing liver injury [15]. The compound Ca-074 methyl ester (Ca-074 Me), a selective inhibitor of cathepsin B, has been demonstrated to exert protective effects within a guinea pig model of polymyositis precipitated by infection with Coxsackie virus B1 (CVB1) [16]. However, it remains unknown whether cathepsin B may influence cardiomyocyte apoptosis and contribute to the pathogenesis of myocardial injury. Further investigation is required to elucidate the potential mechanistic role of cathepsin B in the regulation of cardiac cell death and the subsequent development of myocardial damage.

## 2 Materials and methods

### 2.1 Mice

Male C57BL/6J mice (12 weeks, 22–25 g) were purchased from the Laboratory Animal Center of Southern Medical University (Guangzhou, China). The mice were housed under a specific pathogen-free condition, on a 12-h light-dark cycle, and with food and water *ad libitum*.

### 2.2 Hypoxia-induced myocardial I/R

Mice were randomly assigned to four groups (*n* = 6 per group) according to whether Ca-074 Me (50 mg/kg) was injected intraperitoneally simultaneously and whether myocardial I/R surgery was performed or not. At the beginning of the procedure, the mice were given an intraperitoneal injection of 50 mg/kg chloral hydrate. Then, an incision of about 1.2 cm was made over the left chest, and a purse suture was made. The fourth intercostal space was exposed after dissection and retraction of the pectoral major and minor muscles. A small hole was made at the fourth intercostal space with a mosquito clamp to open the pleural membrane and pericardium. With the clamp open, the heart was popped out through the incision. Locating the left anterior descending (LAD) branch, then using a slipknot tied around the LAD 2–3 mm from its origin with 8–0 silk suture. The heart is then quickly placed back into the thoracic space, followed by manual evacuation of air and the skin closing. The internal needle end of the slipknot suture is cut as short as possible, and the other end of the suture is about 0.8 cm long and remains outside of the chest. After 30 min of ischemia, the slipknot was released by pulling the long end of the slipknot suture smoothly and gently until a feeling of release was sensed; meanwhile, the myocardium began reperfusion. Blood and heart samples were collected after 24 h of reperfusion. Submandibular blood samples (about 200 µl) were obtained by incising the right submandibular vein of mice (22–25 g) with a sterile 4-mm lancet. The animals were euthanized with 4% isoflurane inhalation followed by cervical vertebra dislocation. Animal death was confirmed by respiratory and cardiac arrest and no righting reflex.

### 2.3 Cell culture and stimulation conditions

AC16 cells were cultured in DMEM/F12 (Thermo Fisher Scientific, Carlsbad, CA, USA, A4192001) supplemented with 100 U/ml penicillin, 100 μg/ml streptomycin, and 10% fetal bovine serum at 37°C in a 5% CO_2_ incubator. The cells were treated with different concentrations of CoCl_2_ (0, 10, 50, 100, 200, and 500 µmol/l) or cultured in a hypoxia environment (1% oxygen, 5% carbon dioxide, and 94% nitrogen) for 0, 1, 3, 6, 12, and 24 h to determine the suitable hypoxia stimulation condition. Next, the cells were treated with CoCl_2_ (200 μM, Sigma-Aldrich, 232696) or 1% O_2_ in the presence or absence of Ca-074 Me (10 μM, Merck, S7420). AC16 cells were also treated with Ca-074 Me (10 μM) in combination with or without Z-DEVD-FMK (20 μM, Selleck, S7312) for 24 h under hypoxia.

### 2.4 Generation of gene knockout cells with CRISPR/Cas9

According to the manufacturer’s instructions, AC16 cells were transfected with Cathepsin B CRISPR/Cas9 KO plasmid (Santa Cruz Biotechnology) using UltraCruz^®^ transfection reagent (Santa Cruz Biotechnology). Twenty-four hours after transfection, the expression level of cathepsin B in the cells was assessed by immunoblotting analysis.

### 2.5 Plasmid overexpression

Plasmid was synthesized by the Beijing Genomics Institution (Shenzhen, China) and was used to overexpress cathepsin B expression in HK-2 cells. Before other indicated experiments, the AC16 cells were seeded into six-well plates and were transfected with 10 μg of plasmid with Lipofectamine 3000 reagent (Thermo Fisher Scientific) following the standard protocol provided by the manufacturer.

### 2.6 ELISA assay

Mice’s blood samples were obtained from the carotid artery and centrifuged at 4,000 × *g* for 15 min, and then, the supernatant was collected and set aside at −80°C for serum cytokine analysis. Cytokine levels in the sera were assessed using commercial ELISA kits purchased from eBioscience (San Diego, CA, USA).

### 2.7 Histological evaluation

Heart samples were collected from mice, then fixed in 4% paraformaldehyde, embedded in paraffin, and stained with hematoxylin and eosin (H&E) according to the manufacturer’s instructions. The quantification analysis of H&E staining was established based on the methods of a previous study [17]. In brief, vascular congestion, interstitial capillary damage, vascular dilatation, myocytolysis, and myocyte vacuolization were assessed and rated as minimum (1), mild (2), moderate (3), or severe (4) on a scale of 1–4, with papillary muscle involvement serving as the criterion. Histopathologic changes were graded as minimal (1) if less than 30% of the heart muscles (left and right ventricles, and interventricular septum) were affected without papillary muscle involvement; mild (2) if more than 30% of the heart muscles were affected without papillary muscle involvement; moderate–mild (3) if papillary muscle involvement was focal; and severe (4) if the heart muscle and papillary muscle involvement was diffuse. A scale from 1 to 3 was used to grade myocardial hypertrophy. Mild myocardial hypertrophy was defined as minimal reactive cellular hypertrophy of the left ventricle (1), moderate myocardial hypertrophy as reactive cellular hypertrophy of the left and right ventricles, as well as the interventricular septum (2), and severe myocardial hypertrophy as reactive cellular hypertrophy involving all heart muscles, including papillary muscles with obvious luminal narrowing (3). Scores of 1 and 2 indicated focal or diffuse intravascular inflammation, respectively. Focal intravascular inflammation was defined as dispersed inflammation within interstitial vessels without papillary muscle involvement. A pathologist carried out all scoring in a blinded fashion. For immunohistochemical staining, antigen retrieval was performed in a citrate buffer (pH 6.0) at 120°C for 10 min, and endogenous peroxidase activity was blocked by exposure to 3% H_2_O_2_ for 15 min. Sections were then incubated with primary antibodies at 4°C overnight. Immunoreactivity was detected using the corresponding HRP-conjugated secondary antibody and visualized using a diaminobenzidine kit (Beyotime Biotechnology, Shanghai, China).

### 2.8 Western blot analysis

Protein lysate was prepared in RIPA buffer (Beyotime, Hangzhou, China) supplemented with protease and phosphatase inhibitor cocktails (Beyotime). Protein samples were fractionated by sodium dodecyl sulfate–polyacrylamide gel electrophoresis and electrophoretically transferred onto polyvinylidene fluoride membranes (Millipore, Billerica, MA, USA). After blocking with bovine serum albumin (5%) for 1 h at room temperature, the membranes were incubated overnight at 4°C with primary antibodies. Subsequently, the membranes were incubated with the horseradish peroxidase-conjugated corresponding secondary antibody for 1 h at room temperature. The blots are cut to the appropriate size prior to hybridization with antibodies. Finally, target protein detection was conducted with enhanced chemiluminescence (Tanon, Shanghai, China) according to the manufacturer’s protocol.

### 2.9 RNA isolation and quantitative real-time PCR

Total RNA was isolated from primary astrocytes using TRIzol reagent (Thermo Fisher Scientific, USA) according to the manufacturer’s instructions. Total RNA was used to synthesize cDNA using a PrimeScript RT reagent Kit with gDNA Eraser (TaKaRa, China). Expression of mRNA was determined by quantitative real-time PCR (q-PCR) using the TB Green Premix Ex Taq II (TaKaRa, China). q-PCR was performed on the ABI QuantStudio 6 flex (Applied Biosystems, USA). GAPDH expression was quantified as an internal control for mRNA analysis. The primer sequences used in q-PCR were as follows: GAPDH mRNA-forward: 5′-TTG TCA TGG GAG TGA ACG AGA-3′; GAPDH mRNA-reverse: 5′-CAG GCA GTT GGT GGT ACA G-3′. Cathepsin B mRNA-forward: 5′-TTG CGT TCG GTG AGG ACA TAG-3′; Cathepsin B mRNA-reverse: 5′-GCA GGA GCC CTG GTC TCT A-3′.

### 2.10 Flow cytometry analysis

Cell apoptosis was evaluated using flow cytometry analysis. AC16 cells were incubated in complete DMEM/F12 with or without Ca-074 Me (10 µM) and treated with CoCl_2_ (200 µM) for 24 h at 37°C, or hypoxia chamber for 24 h. For apoptosis quantification by annexin V, AC16 cells were washed with phosphate-buffered saline and subsequently incubated for 5 min at room temperature in the dark in 500 μl of 1× binding buffer containing 5 μl of Annexin V-PE and 10 μl of 7-AAD (Multi Sciences, Hangzhou, China). The cells were acquired and analyzed using the BD FACSDiva program in the flow cytometry FACS LSRFortessa (BD Biosciences, San Jose, CA, USA).

### 2.11 Statistical analysis

All values were expressed as mean ± standard deviation. One-way analysis of variance was used for comparisons among multiple groups. Differences between the two groups in the experiments were analyzed by Student’s *t*-test. A value of *p* < 0.05 was considered statistically significant.


**Ethical approval:** All animal experiments in this study were approved by the Welfare and Ethical Committee for Experimental Animal Care of Southern Medical University (Approval No: L2016014). This study was conducted in accordance with relevant guidelines and regulations.

## 3 Results

### 3.1 Increased cathepsin B levels correlate with myocardial I/R injury model mice

**Figure 1:**
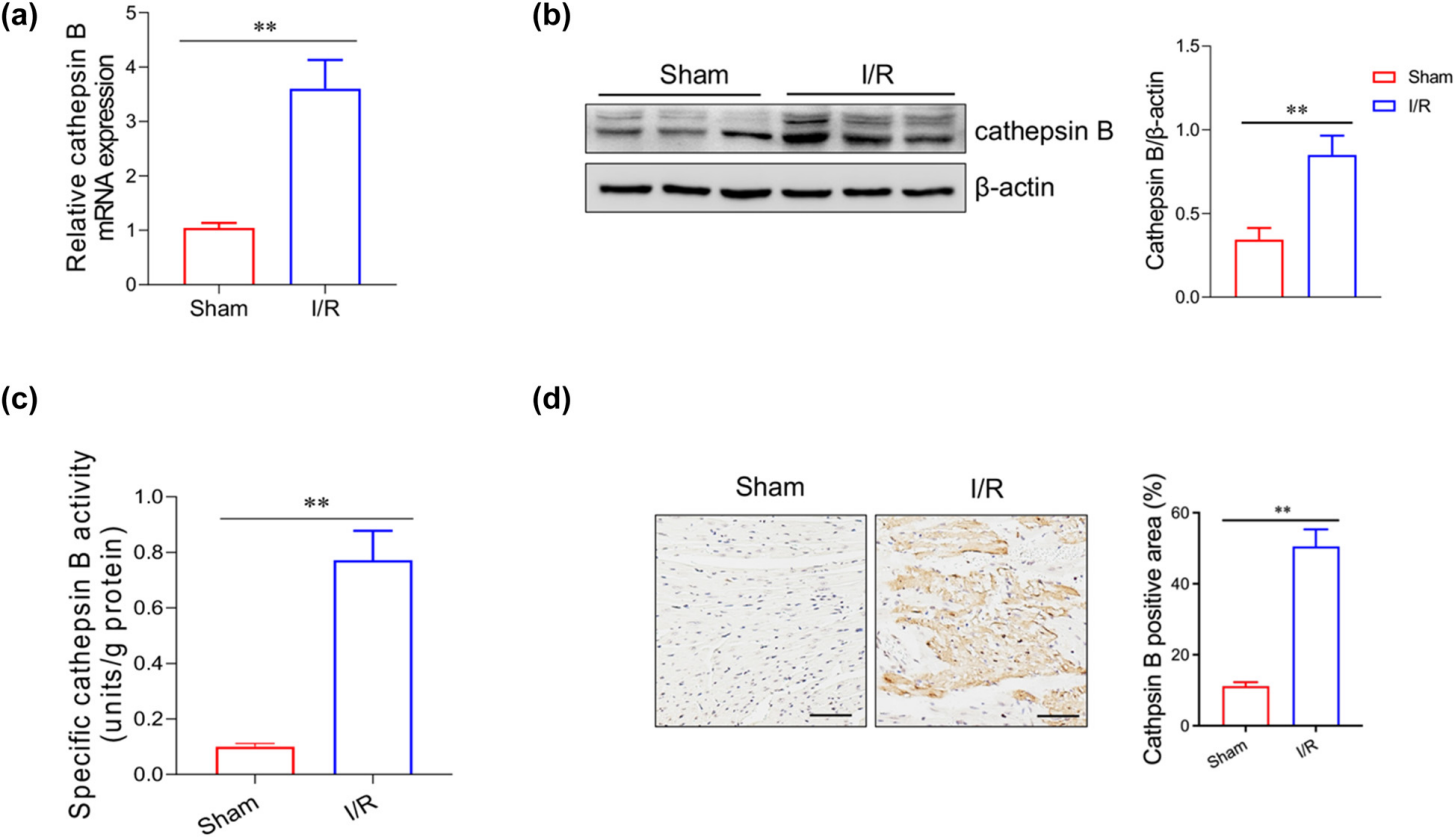
Cathepsin B levels were increased in the heart tissue of myocardial I/R mice. (a) The relative mRNA expression level of cathepsin B in the heart tissue of the myocardial I/R mice was analyzed by qRT-PCR. The protein level (b) and the activity (c) of cathepsin B were evaluated by western blotting analysis and ELISA, respectively. (d) The localization of cathepsin B in the mouse heart tissue was determined by immunohistochemistry. **p < 0.01. Data are representative of three independent experiments with similar results.

To investigate the relationship between cathepsin B and myocardial injury, we developed an I/R-induced myocardial injury mouse model, with a sham group as the control. The mRNA level of cathepsin B of the heart in the I/R group was significantly higher than that in the sham group (Figure 1a). Western blot analysis further confirmed that the cathepsin B expression level in the I/R group was increased compared with the sham group (Figure 1b). We also examined the activity of cathepsin B in both I/R and sham mice. The data revealed a significant elevation of cathepsin B activity in the heart tissue of I/R-treated mice (Figure 1c). Besides, immunohistochemical staining for cathepsin B revealed a higher amount of cathepsin B expression in the myocardial tissues from the I/R group than that from the sham group (Figure 1d). These findings suggest that cathepsin B is closely associated with myocardial injury in mice.

### 3.2 Inhibition of cathepsin B lessens the severity of myocardial injury in mice

**Figure 2:**
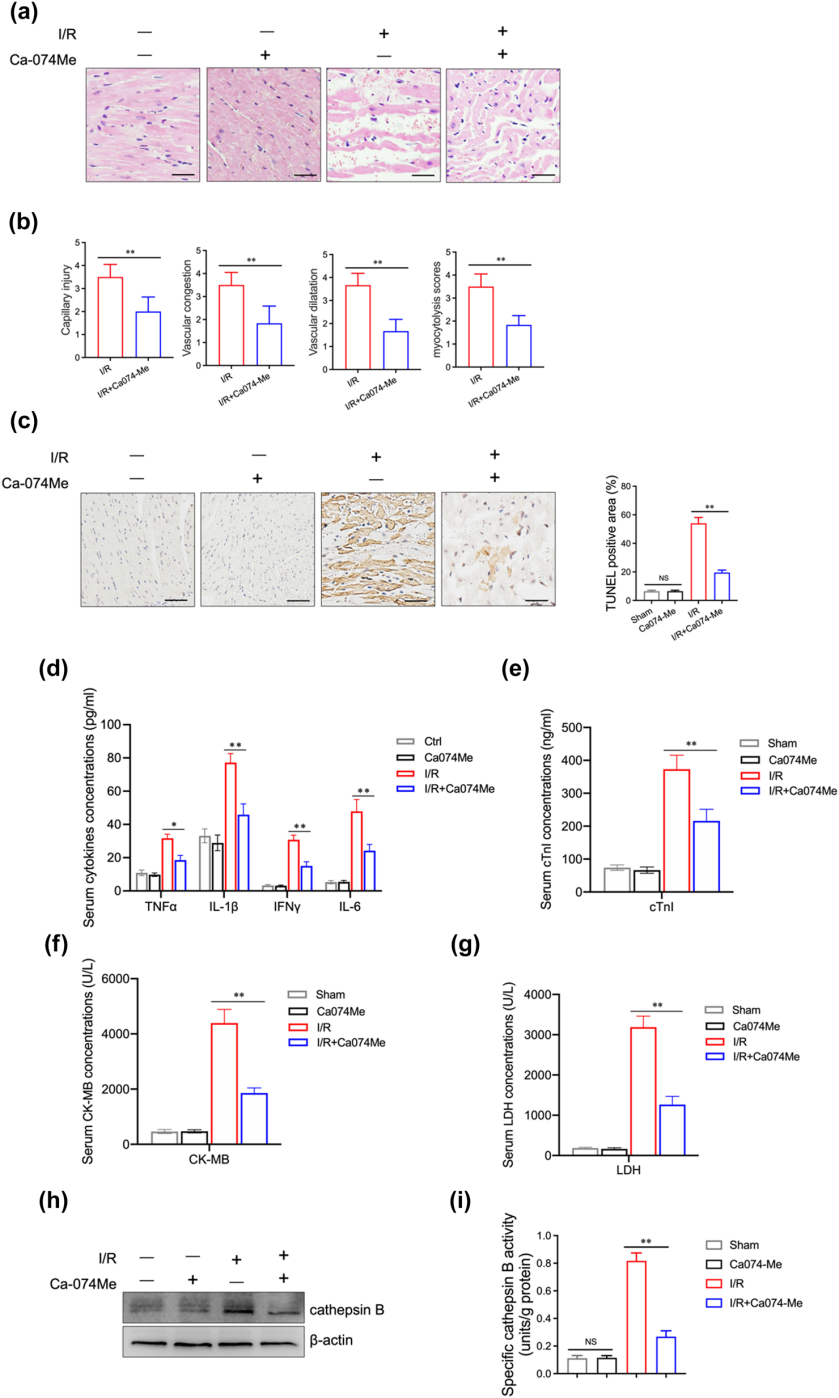
Effects of cathepsin B inhibitor on myocardial injury in myocardial I/R mice. Myocardial ischemia/reperfusion surgery and intraperitoneal injections of Ca-074 Me (50 mg/kg) were simultaneously used to treat mice (*n* = 6 per group). (a) The histological analysis of the mouse heart on day 1 after surgery was performed by H&E. (b) Capillary injury, vascular congestion, vascular dilatation, and myocytolysis scores of myocardial injury were evaluated. (c) Cell death in the mouse heart tissue was examined by TUNEL staining. Scale bar = 100 μm. (d) The expression level of inflammatory cytokines (TNF-α, IL-1β, IFN-γ, IL-6) in serum was measured by ELISA. The levels of cTnl (e), CK-MB (f), and LDH (g) were detected by ELISA. The protein level (h) and the activity (i) of cathepsin B were evaluated by western blotting analysis and ELISA, respectively. *p < 0.05 and **p < 0.01. Data are representative of three independent experiments with similar results.

To clarify the role of cathepsin B in myocardial injury, we pretreated mice with Ca-074 Me, a specific inhibitor of cathepsin B, before I/R treatment. H&E staining showed that the cardiomyocytes were arranged disorderly and enlarged in I/R treated mice. Meanwhile, the infiltration of inflammatory cells was also observed. After Ca-074 Me treatment, the degree of myocardial injury was significantly alleviated compared with the I/R group (Figure 2a). Ca-074 Me can also reduce the area of heart infarction upon hypoxia (Figure 2b). TUNEL staining showed a higher proportion of apoptotic cells in the infarcted area of the I/R group, which was significantly alleviated by Ca-074 Me (Figure 2c). Moreover, inflammatory cytokine levels (IL-1β, IL-6, TNF-α, and IFN-γ) were significantly lower in the Ca-074 Me-treated I/R group compared to the untreated I/R group (Figure 2d). Additionally, Ca-074 Me can reduce the levels of CK-MB, cTnl, and LDH in serum (Figure 2e–g). The data suggest that cathepsin B may modulate the pathogenesis of myocardial I/R injury.

### 3.3 Cathepsin B expression is increased in AC16 cells under hypoxic condition

**Figure 3:**
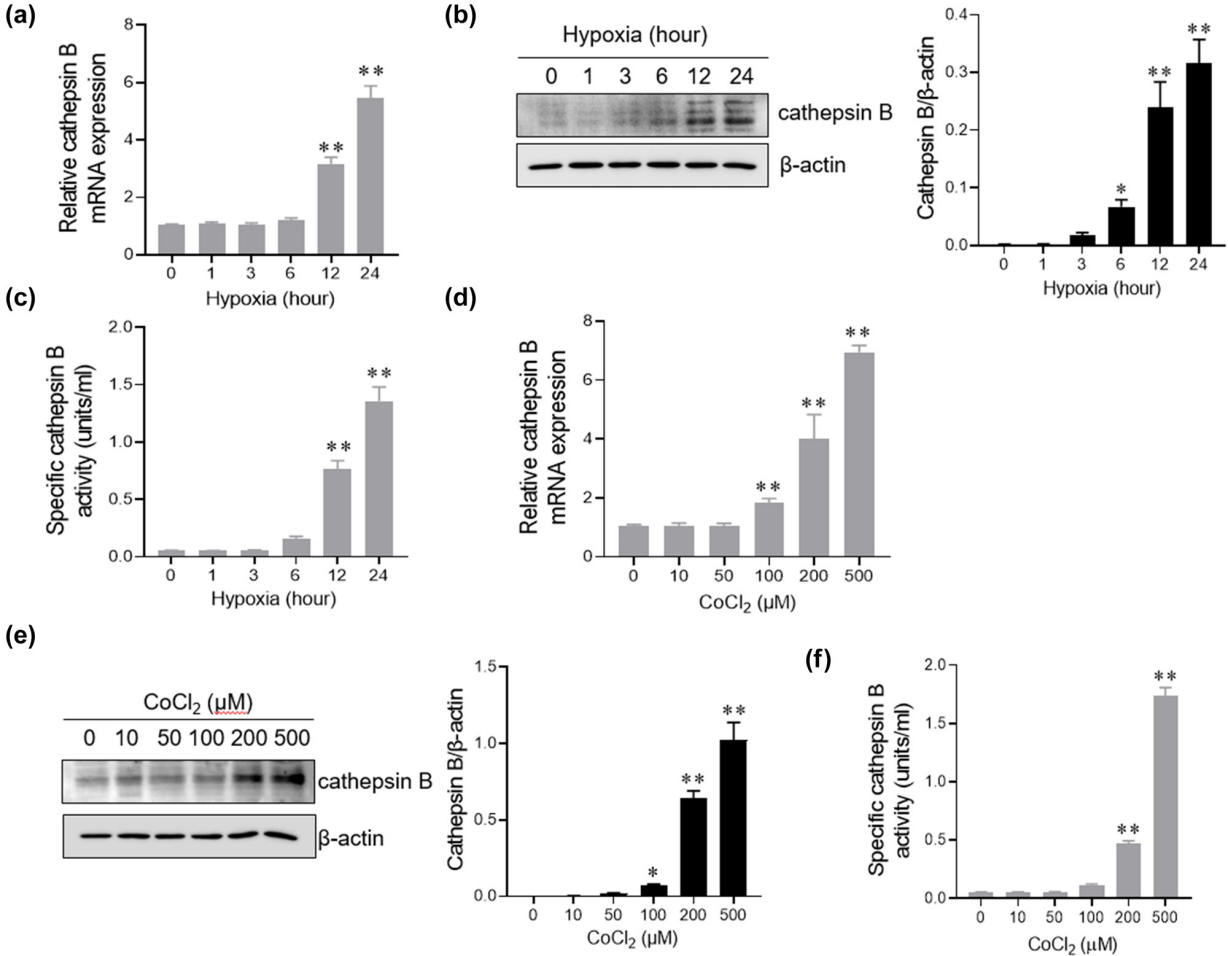
Cathepsin B expression is increased in AC16 cells under hypoxic conditions. (a–c) AC16 cells were incubated with 1% O_2_ for 0, 1, 3, 6, 12, and 24 h. The mRNA level of cathepsin B was analyzed by q-PCR (a); the protein level of cathepsin B was evaluated by western blotting analysis (b); and the activity of cathepsin B was assessed by ELISA (c). (d–f) AC16 cells were treated with different CoCl_2_ concentrations (0, 10, 50, 100, 200, and 500 μM) for 24 h. The mRNA level of cathepsin B was analyzed by q-PCR (d). The protein level of cathepsin B was evaluated by western blotting analysis (e). The activity of cathepsin B was assessed by ELISA (f). **p < 0.01. Data are representative of three independent experiments with similar results.

Myocardial cell damage is the leading cause of cardiac I/R injury [18]. Hypoxia is an essential factor in causing myocardial damage. To investigate whether cathepsin B release is related to hypoxia, we used the human myocardial cell AC16 cells to be cultured in the 1% O_2_ condition and Cocl_2_-induced hypoxic environment at different times. The mRNA and protein levels of cathepsin B significantly increased after incubation with 1% O_2_ (Figure 3a–c) or treatment with varying concentrations of CoCl_2_ (Figure 3d–f). Furthermore, the activity of cathepsin B was also increased in a time- and dose-dependent manner under hypoxic conditions. Above all, these data indicate that cathepsin B expression was increased in myocardial cells under hypoxia stimulation.

### 3.4 Inhibition of cathepsin B reduces AC16 cells’ apoptosis under hypoxic condition

**Figure 4:**
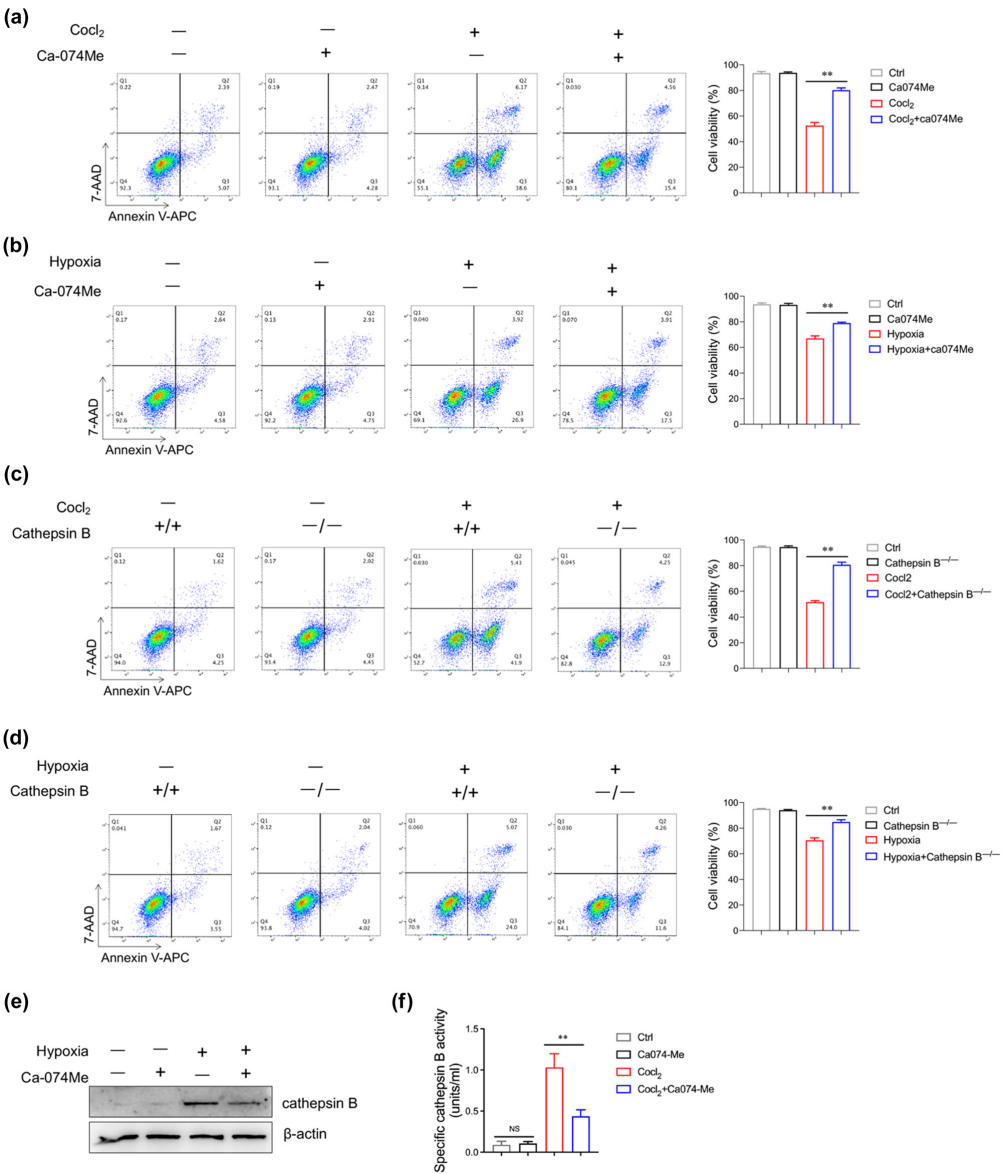
Inhibition of cathepsin B reduces AC16 cell apoptosis under hypoxic conditions. (a) AC16 cells were treated with CoCl_2_ (200 μM) in combination with or without Ca-074 Me (10 μM) for 24 h. Cell viability of AC16 cells was evaluated by flow cytometry. (b) AC16 cells were cultured under 1% O_2_ condition in combination with or without Ca-074 Me (10 μM) for 24 h. Cell viability of AC16 cells was determined by flow cytometry. (c) Cathepsin B^+/+^ and cathepsin B^−/−^ AC16 cells were stimulated with CoCl_2_ (200 μM) with or without Ca-074 Me (10 μM) for 24 h, and then, the cell viability of AC16 cells was evaluated by flow cytometry. (d) Cathepsin B^+/+^ and cathepsin B^−/−^ AC16 cells were cultured under 1% O_2_ condition with or without Ca-074 Me (10 μM) for 24 h, and then the cell viability of AC16 cells was determined by flow cytometry. (e and f) AC16 cells were treated with CoCl_2_ (200 μM) in combination with or without Ca-074 Me (10 μM) for 24 h. The protein level of cathepsin B was evaluated by western blotting analysis (e); the activity of cathepsin B was assessed by ELISA (f). **p < 0.01. Data are representative of three independent experiments with similar results.

To further validate the role of cathepsin B in hypoxia-mediated cardiomyocyte survival, AC16 cells were pretreated with a specific cathepsin B inhibitor, Ca-074 Me, before CoCl_2_ or 1% O_2_ treatment. Compared to 1% O_2_ and CoCl_2_ treatment, Ca-074 Me pretreatment significantly reversed the proportion of cardiomyocyte apoptosis (Figure 4a and b). Similarly, cathepsin B deficiency decreased hypoxia-induced cardiomyocyte apoptosis (Figure 4c and d). Previous studies have found that cathepsin B is related to endogenous apoptosis in the process of myocardial remodeling [19]. In addition, exogenous cathepsin B can promote cell apoptosis in a dose-dependent manner (Figure S1). These data suggest that cathepsin B inhibition prevents hypoxia-induced cardiomyocyte apoptosis.

### 3.5 Cathepsin B promotes AC16 cell apoptosis under hypoxic conditions through the caspase-3 pathway

**Figure 5:**
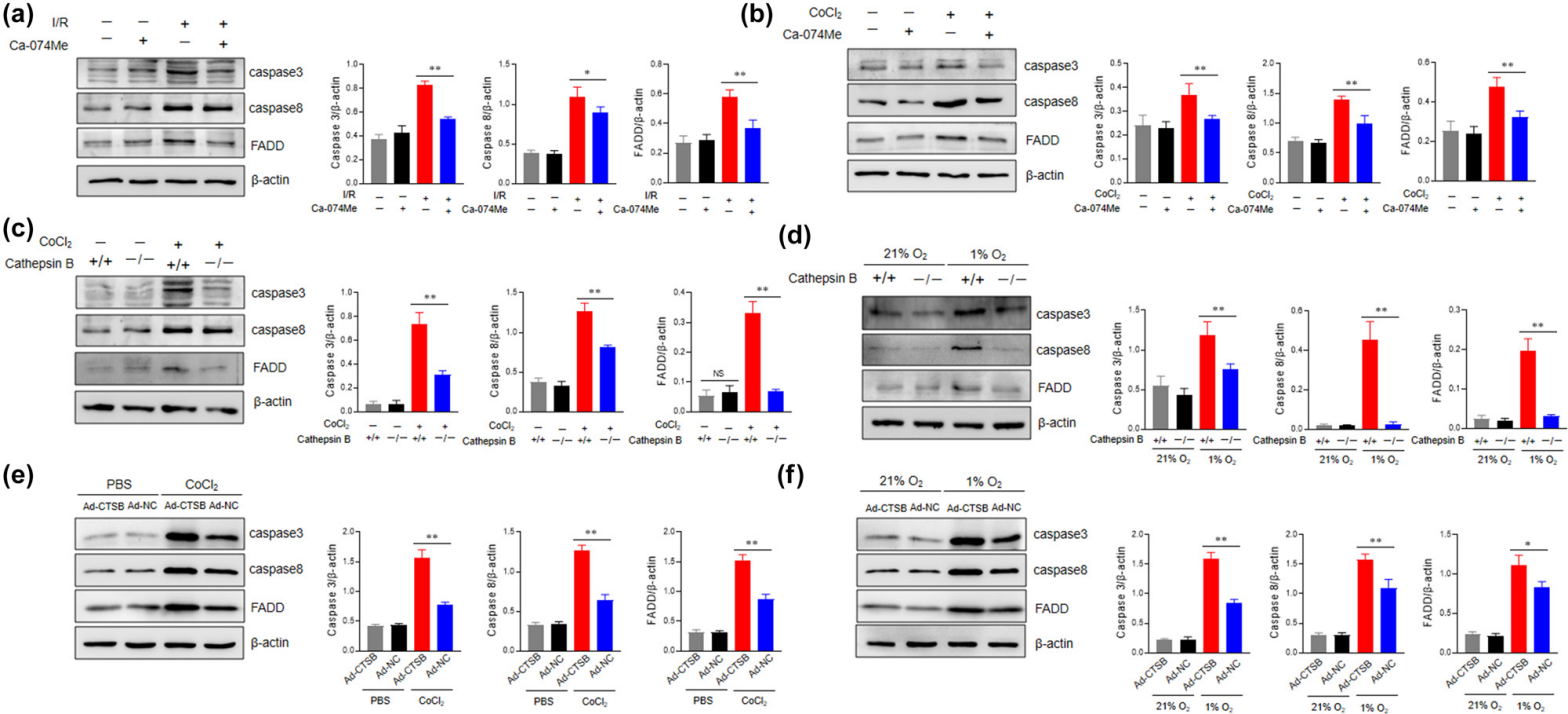
Cathepsin B promotes apoptosis of AC16 cells under hypoxic conditions through the caspase-3 pathway. (a) The protein levels of caspase-3, caspase-8, and FADD in AC16 cells cultured under 1% O_2_ condition with or without Ca-074 Me (10 μM) were evaluated by western blotting analysis. (b) The protein levels of caspase-3, caspase-8, and FADD in AC16 cells stimulated with CoCl_2_ (200 μM) for 24 h with or without Ca-074 Me (10 μM) were evaluated by western blotting analysis. (c) Cathepsin B^+/+^ and cathepsin B^−/−^ AC16 cells were cultured under 1% O_2_ condition with or without Ca-074 Me (10 μM) for 24 h, then the expression of caspase 3, caspase 8, and FADD was determined by western blotting analysis. (d) Cathepsin B^+/+^ and cathepsin B^−/−^ AC16 cells were stimulated with CoCl_2_ (200 μM) for 24 h with or without Ca-074 Me (10 μM), then the expression of caspase 3, caspase 8, and FADD was determined by western blotting analysis. (e and f) AC16 cells were seeded into six-well plates and allowed to grow at 50–70% confluence. The cells were transfected with the expression plasmid of cathepsin B for 48 h. (e) The protein levels of caspase-3, caspase-8, and FADD in AC16 cells stimulated with CoCl_2_ (200 μM) for 24 h were evaluated by western blotting analysis. (f) The protein levels of caspase-3, caspase-8, and FADD in AC16 cells cultured under 21% O_2_ or 1% O_2_ condition were evaluated by western blotting analysis. *p < 0.05, **p < 0.01, ns not significant. Data are representative of three independent experiments with similar results.

To verify the conjecture of cathepsin B with cardiomyocyte apoptosis under hypoxia conditions, the caspase-3 pathway-related proteins were analyzed by western blotting analysis. The expression level of caspase-3/8 and FADD was increased after hypoxia induction and significantly reduced upon Ca-074 Me treatment (Figure 5a and b). Similar results were observed in cathepsin B^–/–^ AC16 cells under hypoxia stimulation (Figure 5c and d). To further confirm the role of caspase 3 in cathepsin B-mediated apoptosis, AC16 cells were pretreated with a specific caspase-3 inhibitor, Z-DEVD-FMK. Of note, the cell viability was similar in AC16 cells treated with Ca-074 Me alone or Ca-074 Me plus Z-DEVD-FMK (Figure S2). Overexpression of cathepsin B significantly elevated apoptosis-related protein levels following hypoxia (Figure 5e and f). In conclusion, we determined that cathepsin B promotes the apoptosis of myocardial cells under hypoxic conditions via activating the caspase-3 pathway. In addition, inhibiting the expression of cathepsin B can alleviate the degree of apoptosis in myocardial cells under hypoxic conditions.

## 4 Discussion

Increased cathepsin gene expression is associated with cardiac stress, remodeling, and dysfunction [20]. In this study, we observed a marked elevation in cathepsin B in murine cardiac tissue in response to I/R-induced myocardial injury. Moreover, our study delineates the beneficial impact of cathepsin B inhibition on myocardial preservation, as evidenced by enhanced cardiomyocyte viability. Concomitantly, cathepsin B inhibition was found to significantly attenuate the induction of pro-inflammatory cytokines in the context of a murine model of cardiac I/R injury. Mechanistic studies further revealed that cathepsin B inhibitor affects cardiomyocyte apoptosis by limiting the activation of the caspase 3 pathway.

Lysosomal proteases are implicated in the pathogenesis of various diseases, including autoimmune, metabolic, cardiovascular, and neurodegenerative conditions [21]. Cathepsins are the major lysosomal proteases localized predominantly in the acidic milieu of endo/lysosomal compartments and are integral to many physiological processes, such as proteolysis, energy metabolism, and immunological responses. Among all the lysosomal proteases, cathepsin B has been identified as a critical agent in lysosomal destabilization and the induction of cellular apoptosis [10,22]. The translocation of cathepsin B into the cytoplasm following lysosomal rupture leads to the initiation of apoptotic signaling cascades. Previous studies demonstrated that cathepsin B contributes to fulminant hepatic failure and acetaminophen hepatotoxicity via hepatocyte apoptosis [23,24]. Furthermore, the involvement of cathepsin B-dependent pathways in TNF-α-mediated hepatocyte apoptosis has been demonstrated by using cathepsin B-deficient mice [25]. This was further validated in a study that pharmacological inhibition of cathepsin B attenuated TNF-α-induced liver injury in mice [26]. Cathepsin B seems to participate in producing pro-apoptotic reactants to protect anti-apoptotic reactants from degradation and removal [14]. Herein, we observed that cathepsin inhibition prevented myocardial I/R injury and cardiomyocyte apoptosis, which is related to reduced caspase 3 activity.

Myocardial cell apoptosis is associated with the production of cytokines that promote inflammation during myocardial injury [4]. We also found that cathepsin B-mediated I/R cardiac damage leads to apoptosis and stimulates the production of pro-inflammatory chemokines. Indeed, cathepsins participate in the production of immune mediators through a limited proteolysis process [9]. Cathepsin B enhances the activation of NLRP3 inflammasome, thereby upregulating IL-1β release from endothelial cells [11]. Also, cathepsin B was required for optimal processing of TNF-α and IL-6 in response to inflammatory stimulation [27,28]. Cathepsin B promotes IκBα degradation and NF-κB nuclear translocation in microglia cells in neonatal mice with hypoxic-ischemic brain injury [29]. Indeed, cathepsin B has been implicated in the pathophysiology of numerous inflammatory diseases and cancer. Therefore, it is interesting to reveal the modulatory function of cathepsin B in the initiation and activation of the signaling pathway related to inflammatory cytokines production, and the role of cathepsin B in the cellular inflammatory response during cardiomyocyte damage remains to be further investigated.

The cardiomyocytes play crucial roles in maintaining myocardial function [30]. Aberrant cardiomyocyte function is linked to the development of myocardial injury. Cardiomyocytes are highly susceptible to apoptosis under hypoxic conditions [31]. Hypoxia induces cellular apoptosis, with the caspase cascade playing a critical role in the induction, transduction, and amplification of intracellular apoptotic signals [32]. Our studies demonstrated that cathepsin B inhibition protects against myocardial injury and hypoxia-induced cardiomyocyte apoptosis. Deng *et al.* observed that hypoxia-ischemia stimulation significantly increases caspase-3 expression and neuronal apoptosis in the brains of neonatal mice [33]. By attenuating the activation of the caspase-3 enzyme, it is possible to impede the progression of apoptosis, thus alleviating the harmful effects of hypoxia-ischemia on cellular integrity [34]. In the Fas signaling pathway, FADD is an essential adaptor protein critical for transmitting apoptotic signals [35]. FADD recruits caspase-8 through interactions of the death effect domain, activating the apical caspase [36]. In the present study, Ca-074 Me treatment significantly decreased the enhanced expression of caspase-3, caspase-8, and FADD in cardiomyocytes under hypoxia conditions. Furthermore, Ca-074 Me treatment reduced the expression of endogenous apoptosis-related proteins in cardiomyocytes under hypoxia conditions. The data suggested that cathepsin B inhibition-mediated prevention of cardiomyocyte apoptosis was associated with the downregulation of caspase activation.

In summary, our study demonstrated that inhibition of cathepsin B mitigates I/R-induced myocardial injury in murine models by preserving cardiomyocyte viability. Mechanistic studies elucidated that cathepsin B inhibition reduces the expression of proteins associated with the apoptotic pathway, thereby enhancing cardiomyocyte survival. Most importantly, we have provided experimental evidence showing a potential therapeutic efficacy of cathepsin B inhibition towards attenuating myocardial injury in mice.

## Supplementary Material

Supplementary Figure
