# Supplementary material for: Suppression of cathepsin B attenuates myocardial injury via limiting cardiomyocyte apoptosis
Source: Open Med (Wars). 2025 Nov 26;20(1):20241115. doi: 10.1515/med-2024-1115 (PMC12658728; doi:10.1515/med-2024-1115)
Supplement: Supplementary Figure [file med-2024-1115-sm.pdf]

Supplementary material

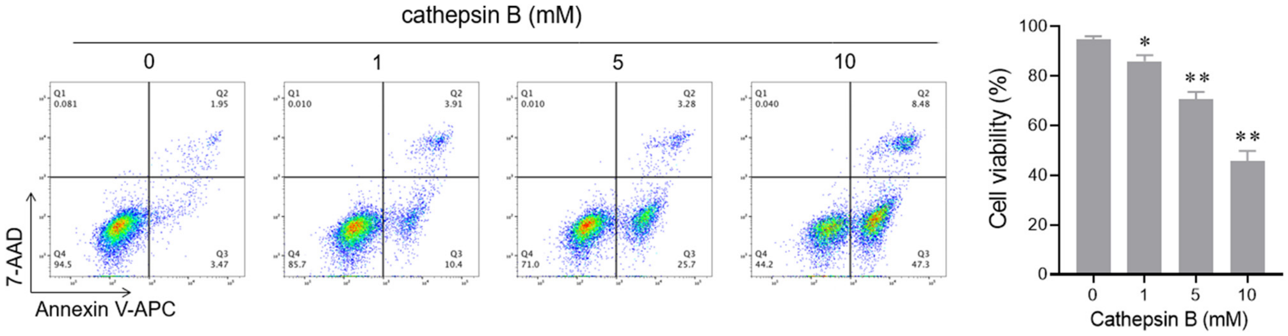

Figure S1: Cathepsin B affects the cell viability of AC16 cells. After treatment with different concentrations of cathepsin B (0, 1, 5, 10 mM), the cell viability of AC16 cells was detected by flow cytometry $*p < 0.05$, $**p < 0.01$. Data are representative of three independent experiments with similar results.

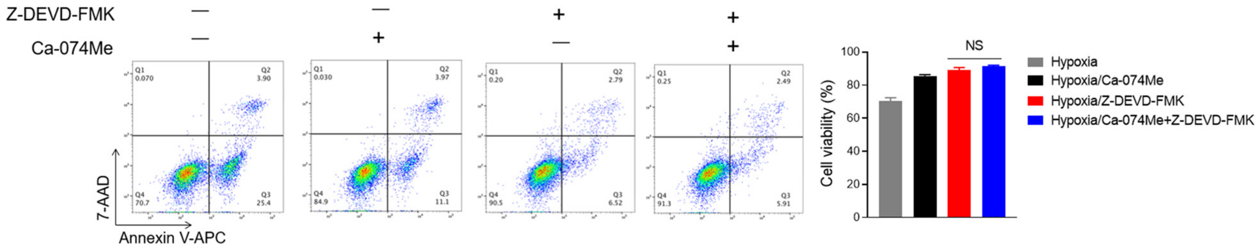

Figure S2: Caspase 3 is involved in the effect of Ca074-Me on cell viability of AC16 cells. AC16 cells were treated with Ca-074 Me in combination with or without Z-DEVD-FMK (20 μM) for 24 h under hypoxia condition. Cell viability of AC16 cells was detected by flow cytometry. NS, not significant. Data are representative of three independent experiments with similar results.
